# Clinical Insights into Mitochondrial Neurodevelopmental and Neurodegenerative Disorders: Their Biosignatures from Mass Spectrometry-Based Metabolomics

**DOI:** 10.3390/metabo11040233

**Published:** 2021-04-10

**Authors:** Haorong Li, Martine Uittenbogaard, Ling Hao, Anne Chiaramello

**Affiliations:** 1 Department of Chemistry, George Washington University, Science and Engineering Hall 4000, 800 22nd St., NW, Washington, DC 20052, USA; haorong@gwmail.gwu.edu; 2 Department of Anatomy and Cell Biology, School of Medicine and Health Sciences, George Washington University, 2300 I Street N.W. Ross Hall 111, Washington, DC 20037, USA; mbogaard@gwu.edu

**Keywords:** mitochondrial genetics, neurometabolic coupling, mitochondrial neurodevelopmental disorders, secondary mitochondrial neurodegenerative diseases, mass spectrometry, metabolomics

## Abstract

Mitochondria are dynamic multitask organelles that function as hubs for many metabolic pathways. They produce most ATP via the oxidative phosphorylation pathway, a critical pathway that the brain relies on its energy need associated with its numerous functions, such as synaptic homeostasis and plasticity. Therefore, mitochondrial dysfunction is a prevalent pathological hallmark of many neurodevelopmental and neurodegenerative disorders resulting in altered neurometabolic coupling. With the advent of mass spectrometry (MS) technology, MS-based metabolomics provides an emerging mechanistic understanding of their global and dynamic metabolic signatures. In this review, we discuss the pathogenetic causes of mitochondrial metabolic disorders and the recent MS-based metabolomic advances on their metabolomic remodeling. We conclude by exploring the MS-based metabolomic functional insights into their biosignatures to improve diagnostic platforms, stratify patients, and design novel targeted therapeutic strategies.

## 1. Introduction

### 1.1. Mitochondria Function as Metabolic Nodes

**Figure 1:**
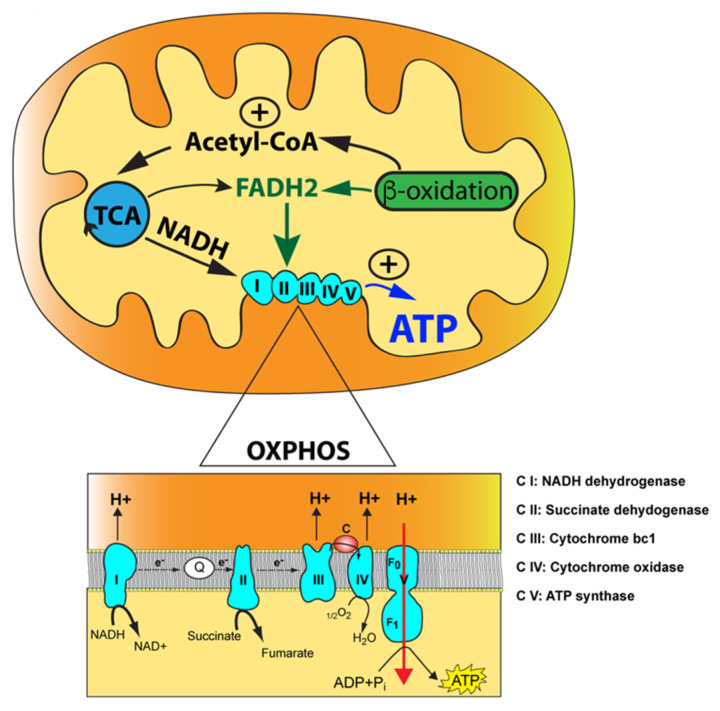
The mitochondrial oxidative phosphorylation (OXPHOS) enzymatic machinery and key metabolic pathways. The mitochondrion is illustrated with its outer mitochondrial membrane, inner mitochondrial membrane, and mitochondrial matrix (yellow) along with the tricarboxylic acid (TCA) cycle and the fatty acid beta-oxidation with their functional link via acetyl-CoA. The reducing agents, NADH and FADH2, produced by the tricarboxylic acid (TCA) cycle and fatty acid beta-oxidation, are indicated along with their two points of entry in the OXPHOS pathway. Magnification of the OXPHOS enzymatic machinery with electrons transfer via the two-electron carriers, coenzyme Q10 (Q) and cytochrome c (c), is shown below. The electron donor, succinate, a product of the TCA cycle, is also indicated acting at the level of complex II. Finally, protons translocated from complexes I, III, and IV into the intermembrane space (orange) are shown along with protons being transferred back into the mitochondrial matrix via complex V, resulting in ATP synthesis from ADP and inorganic phosphate (Pi).

Mitochondria are ubiquitous double membraned multitask organelles where energy is generated through aerobic respiration. Mitochondria produce the majority of ATP required for cellular functions via the oxidative phosphorylation (OXPHOS) pathway, a process involving a flow of electrons through a series of multisubunit OXPHOS complexes, also known as the electron transfer chain (ETC) (Figure 1).

OXPHOS is interlinked with the tricarboxylic acid (TCA) cycle that produces the reducing equivalents, NADH and FADH2, to feed electrons to complex I (NADH dehydrogenase) and complex II (succinate dehydrogenase) of the ETC, respectively (Figure 1). Electrons are then transferred to complex III (ubiquinol cytochrome c oxidoreductase) via ubiquinone, the reduced form of coenzyme Q (CoQ), and subsequently to complex IV (cytochrome c oxidoreductase) via the electron carrier cytochrome c where O_2_ functions as an electron acceptor to produce water in the presence of hydrogen. The flow of electrons through the ETC is coupled with the proton motor force formed by complexes I, III, and IV. This gradient of protons is converted by complex V (ATP synthase) into chemical energy for ATP synthesis using ADP and inorganic phosphate. Acetyl-CoA occupies a central and pivotal position as a key metabolic intermediate for mitochondrial energy metabolism. It is generated by several pathways: (1) oxidation of pyruvate generated during glycolysis; (2) fatty acid oxidation; and (3) oxidative degradation of the amino acids, leucine, isoleucine, and tryptophan (Figure 1) [1,2]. This convergence of pathways highlights the concept of mitochondria operating as hubs for additional biochemical pathways, such as heme biosynthesis, nucleotide biosynthesis (pyrimidines and purines), steroid hormone biosynthesis, calcium homeostasis, innate immunity, and cell death programming [3,4].

### 1.2. Mitochondrial Inheritance

Mitochondria originate from a symbiotic event during which an archaebacterium engulfed a proteobacterium resembling a modern-day rod-negative bacterium with aerobic metabolism capacity via an ETC encoded by its own genome. This event occurred once 1.5 billion years ago and led to the modern-day eukaryotic cells with mitochondria retaining their own circular genome of limited size (about 16,569 bp) and coding capacity, after a phenomenon called reductive evolution. The mitochondrial genome contains 37 genes encoding 2 mitochondrial ribosomal RNAs (mt-rRNAs), 22 mitochondrial tRNAs (mt-tRNAs) and 13 proteins, all of them required subunits of the OXPHOS complexes I, III, IV, and V. Thus, the nuclear genome is a required major contributor to mitochondrial functions via 1158 nuclear-encoded proteins [5,6,7].

The human mitochondrial genome has an overall uniparental inheritance, with the maternal genome being the only one passed on to progeny [8]. However, several species, such as Drosophila, mouse and sheep, exhibit leakage of the paternal mitochondrial genome [9,10,11,12]. Following fertilization of the human oocyte, the paternal mitochondrial genome is eliminated via elusive mechanisms. Recent studies have provided several breakthroughs in elucidating these molecular mechanisms. In *Caenorhabditis elegans*, paternal mitochondria lose their inner membrane integrity due to the relocation of its mitochondrial endonuclease G, CPS-6, from the intermembrane space to the mitochondrial matrix, resulting in degradation of paternal mitochondrial DNA (mtDNA) [13]. In *Drosophila melanogaster*, paternal mtDNA transmission is eliminated through a multistep mechanism [14]. During spermatogenesis, the paternal mitochondrial genome undergoes a drastic elimination via the action of the nuclear-encoded mitochondrial DNA polymerase (polγ−α) *tamas* [15]. After fertilization, sperm-derived mitochondria are degraded through endocytotic and autophagic processes mediated by the ubiquitin–proteasome system [16]. In humans, the precise mechanism responsible for the active elimination of paternal mtDNA remains to be elucidated. A recent study has highlighted the leakage of paternal mtDNA in three unrelated multigeneration families [17]. Despite this paternal leakage, the predominant mitochondrial genome in the fertilized oocyte remains maternal, with the paternal mitochondrial genome present at extremely low levels that are only detectable by in-depth sequencing all the copies of the mitochondrial genome [18]. Uniparental inheritance of mtDNA is postulated to be a protective mechanism by which fertilized oocytes minimize the introduction of paternal mtDNA variants. During preimplantation stages, the fertilized oocyte relies on the 100,000 maternally inherited mitochondria to undergo early embryonic development, as mitochondrial biogenesis only occurs at the onset of organogenesis [19]. This progressive reduction in mitochondrial number until neurogenesis is referred to as the bottleneck theory, a mechanism that protects cells or individuals against the extensive burden of mitochondrial variants, underscoring the interest of uniparental mitochondrial inheritance [20,21]. Lending further support to the theory of “genome compatibility” between the mitochondrial and nuclear genomes is the phenotype of engineered mouse lines containing both parental mitochondrial genomes [22]. Their altered metabolism, impaired cognitive function, and elevated stress levels support the concept of optimal matching between mitochondrial and nuclear genomes for efficient OXPHOS [22].

### 1.3. Mitochondrial Copy Number and Heteroplasmy

The mitochondrial genome is present in multiple copies per cell. However, the mtDNA copy number greatly varies among cell types to adapt to their cellular bioenergetic needs. The unfertilized oocyte has 500,000 copies of mtDNA, whereas the sperm only has a few hundred copies [23]. In contrast, the red blood cells have no mtDNA. MtDNA copy number is a quantifiable biomarker of mitochondrial fitness, as it is directly correlated to mitochondrial biogenesis, mitochondrial transcription and translation [24]. Suboptimal mtDNA levels result in decreased OXPHOS and ATP levels, leading to mitochondrial dysfunctions. Furthermore, the altered mtDNA copy number is often associated with key pathological changes during aging and disease progression [25]. Thus, mtDNA copy number is tightly coordinated and regulated in health and disease. However, its precise underpinning mechanisms remain poorly understood.

The mtDNA copy number is altered by environmental stressors, therapeutic drugs, and oxidative stress, possibly due to its close proximity to the OXPHOS machinery [26]. The principal source of oxidative stress comes from the OXPHOS process that produces by-products of reactive oxygen species (ROS), such as hydrogen peroxide (H_2_O_2_), hydroxyl (OH^•^), and superoxide (O_2_^−^). Enzymatic activities of complexes I and III generate low levels of O_2_^−^, which is converted into H_2_O_2_ via superoxide dismutase (SOD) 1 or 2 in the intermembrane space and mitochondrial matrix, respectively [27]. MtDNA is not vulnerable to O_2_^−^ or H_2_O_2_ but rather to the conversion of H_2_O_2_ into a highly reactive hydroxyl radical HO^•^ through Fenton chemistry in the presence of free iron (Fe^2+^). When they are produced at a higher rate than the antioxidant capacity of the cell’s arsenal of ROS mitochondrial scavenger proteins, oxidative damage occurs, leading to the accumulation of somatic mitochondrial variants and altered mitochondrial metabolic functions. These mtDNA lesions can be repaired via the mitochondrial base excision repair system, a repair system that becomes faulty during aging, resulting in the accumulation of somatic mitochondrial variants. Illustrating this phenomenon is our recent study showing that mtDNA isolated from a 58-year-old mother exhibits a higher number of somatic mitochondrial variants than that of her 24-year-old daughter [28]. Regardless of whether they are causal or correlative, they appear to be strong contributors to the aging phenotype [29].

**Figure 2:**
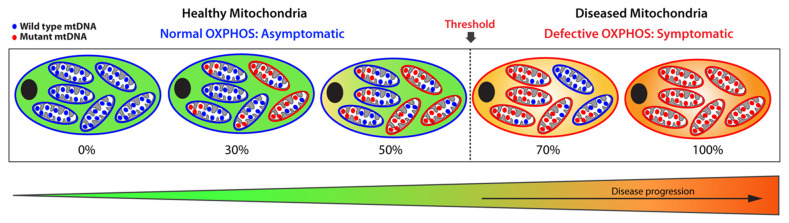
Schematic representation of heteroplasmic threshold. Healthy (functional) mitochondria and wild-type mtDNA are illustrated in blue, while diseased (dysfunctional) mitochondria and mutant mtDNA are shown in red. When a population of diseased mitochondria is below a cell-specific heteroplasmic threshold, it ensures normal overall OXPHOS activity and, therefore, an asymptomatic status. Conversely, when the population of diseased mitochondria exceeds the heteroplasmic threshold tolerated by a cell, it results in defective OXPHOS, giving rise to a symptomatic status. Thus, mitochondrial heteroplasmy modulates disease progression.

Somatic or most inherited mitochondrial variants only affect a subset of the multi-copy mitochondrial genome resulting in heteroplasmy, which is defined as the co-existence of wild-type (WT) and mutated mtDNA population within a mitochondrion (Figure 2) [30].

The amount of mutant mtDNAs dictates the fitness of a mitochondrion: when the load of mutated mtDNA exceeds a certain threshold where WT mtDNA can no longer compensate, the mitochondrion becomes dysfunctional due to defective OXPHOS, leading to severe and debilitating phenotypic manifestations. Below that threshold, the mitochondrion remains functional with sufficient OXPHOS capacity. This biochemical threshold varies among cell and tissue type, as well as organs, and oscillates between 60% to 90%, a range determined by the bioenergetic need of a cell or organ and by the type of the mitochondrial variants. Above that threshold, levels of heteroplasmy broadly correlate with disease severity. By contrast, homoplasmy refers to a pathogenic mitochondrial variant present on all the mtDNA copies, which leads to the most severe phenotypic manifestations. These homoplasmic variants are rare and exhibit a curtailed clinical penetrance.

Factors modulating the penetrance of a pathogenic mitochondrial variant remain poorly understood [31]. Most of the acquired somatic mitochondrial variants have low levels of heteroplasmy, which could only be unmasked with the advent of next-generation sequencing. Long-range PCR followed by massively parallel sequencing accurately and reliably quantifies low heteroplasmic levels with a high level of confidence by applying a stringent threshold [32]. Healthy individuals harbor multiple somatic mitochondrial variants with very low heteroplasmy as a result of lifetime accumulation of somatic oxidative DNA damages and replication errors. Their influence on neurodegenerative diseases and inherited mitochondrial disorders remains elusive, but their prevalence in healthy individuals supports the concept of universal mtDNA heteroplasmy [33,34].

## 2. Genetics of Mitochondrial Diseases

Mitochondrial dysfunction causes diseases spanning from intractable neonatal neurodevelopmental diseases to adult-onset neurodegenerative diseases. Mitochondrial diseases are classified as primary or secondary [35]. Primary mitochondrial diseases are those with respiratory chain deficiencies resulting in deficient mitochondrial ATP synthesis caused by mitochondrial and/or nuclear variants. In contrast, secondary mitochondrial diseases have a mitochondrial etiology due to nuclear variants mapping in genes encoding proteins involved in mitochondrial functions other than energy metabolism. They are also caused by environmental stressors, certain prescription medications, or a natural decrease in mitochondrial activities due to aging.

### 2.1. Primary Mitochondrial Diseases

Mitochondrial primary diseases are often referred to as mitochondrial respiratory disorders (MRDs) due to their OXPHOS defects. Since 6% of the active nuclear genome is dedicated to mitochondrial functions and their maintenance [36], MRDs exhibit two patterns of inheritance: maternal inheritance for pathogenic mitochondrial variants and a Mendelian inheritance for nuclear variants with an autosomal recessive or dominant pattern, or X-chromosome [37,38]. The prevalence of MRDs caused by nuclear variants is high compared to that of maternally inherited MRDs, which have an estimated prevalence of 1 in 5000 [39]. MRDs are severe multi-systemic diseases with a highly variable clinical heterogeneity and a puzzling genotype–phenotype correlation. Patients harboring the same pathogenic variant can exhibit distinct phenotypic manifestations since the penetrance of the mitochondrial variant is under the influence of phenotypic modulators, such as mitochondrial haplogroup, nuclear background, and epigenetic background [31]. This results in a challenging diagnosis and management of the symptoms [40]. MRDs predominantly affect organs with high-energy demand and dependence on aerobic metabolisms, such as the nervous system and musculoskeletal system, resulting in a constellation of symptoms. Single-organ involvement, such as Leber’s hereditary optic neuropathy (LHON), is rare. The LHON syndrome (OMIM 535000) is caused by one of the three prevalent homoplasmic mitochondrial variants, m.11778G > A, m.3460G > A, and m.14484T > C, resulting in complex I deficiency (Table 1) [41].

Pathogenic mtDNA deletions of mitochondrial single nucleotide variants in genes involved in mitochondrial protein translation or OXPHOS are associated with the most frequent MRDs (Table 1) [31]. Presently, they remain intractable, with only palliative treatments available to patients. The sporadic Kearns–Sayre syndrome (OMIM 530000) is triggered by a single large-scale deletion of 4977 bp of mtDNA, occurring spontaneously in the germline cells (Table 1) [42]. Its late childhood onset is accompanied by primary phenotypic manifestations, such as progressive ophthalmoplegia, pigmentary retinopathy and cardiac conduction defects [43]. Patients can also display secondary clinical symptoms, including ptosis, myopathy, cerebellar ataxia, growth retardation and several endocrinopathies. Mitochondrial encephalopathy, lactic acidosis, and stroke-like episode (MELAS) syndrome (OMIM 540000) is the most common pediatric MRD, which is a progressive multi-system disease mostly caused by the mitochondrial pathogenic variant m.3243A > G mapping in the *MT-TL2* gene encoding the mt-tRNA^Lys (UUR)^ (Table 1) [44]. About 20% of patients harbor other rare mitochondrial single nucleotide variants, including m.1630A > G mapping in the *MT-TV* gene encoding the mt-tRNA^Val^ [28,45], m.3697G > A, m.13514A > G, and m. 14453G > A mapping in the *ND1*, ND5, or *ND6* gene (Table 1) [46,47]. These patients exhibit a complex I deficiency triggering phenotypic manifestations before the age of 20. These include stroke-like episodes, encephalopathy characterized by seizures, lactic acidosis, and myopathy with or without ragged red fibers [48]. This multi-system disease is also associated with additional clinical manifestations, such as cardiomyopathy, gastrointestinal dysmotility, diabetes, neuropathy, hearing impairment, cortical vision loss, ataxia, peripheral neuropathy, learning disabilities, and hemiparesis. However, the frequency of these symptoms greatly varies among MELAS patients, resulting in extreme phenotypic heterogeneity. Maternally inherited Leigh syndrome (MILS; OMIM 516060) is mainly caused by the pathogenic mitochondrial variant m.8993T > G mapping in the *MT-ATPase6* gene encoding the MT-ATPase6 subunit of complex V, responsible for mitochondrial ATP synthesis [49]. This pathogenic variant dysregulates energy reprogramming due to a defective interplay between OXPHOS and glycolysis, triggering a chronic energy crisis [50]. The m.4681C > T and m.13515G > A variants engender complex I deficiency in MILS patients, as they map in the *ND2* or *ND5* mitochondrial gene, respectively (Table 1). MILS Patients not only display the characteristic bilateral lesions of the basal ganglia and brainstem but also a constellation of heterogenous phenotypic manifestations, including developmental delay, psychomotor regression, ataxia, seizures, peripheral neuropathy, and optic atrophy [51]. Like MELAS, MILS is characterized by clinical complexity as a result of excessive clinical heterogeneity.

Mitochondrial diseases due to nuclear variants are the most frequent MRDs, as 1158 human nuclear genes encode mitochondrial proteins [6]. Among them is the most common pediatric MRD, Leigh syndrome, which displays an extensive clinical and genetic heterogeneity with at least 75 monogenic causes mapping in mitochondrial or nuclear genes encoding OXPHOS subunits, factors for OXPHOS assembly, and proteins involved in cofactor biosynthesis and metabolism (Table 1) [43]. OXPHOS deficiency has an early onset spanning from childbirth to early childhood, which is usually unmasked by a viral infection triggering an acute metabolic decompensation. In addition to the cardinal neuropathologic feature of bilateral symmetric lesions in the basal ganglia and brainstem shared by all the Leigh patients, the neurological clinical manifestations are heterogeneous, including encephalopathy, seizures, developmental delay, failure-to-thrive, hypotonia, ataxia, and ophthalmologic abnormalities [44,45]. Such clinical heterogeneity is further exacerbated by the array of non-neurological symptoms exhibited by patients, which affects multiple organs, such as the heart, intestines, liver, kidneys.

### 2.2. Secondary Mitochondrial Diseases

**Figure 3:**
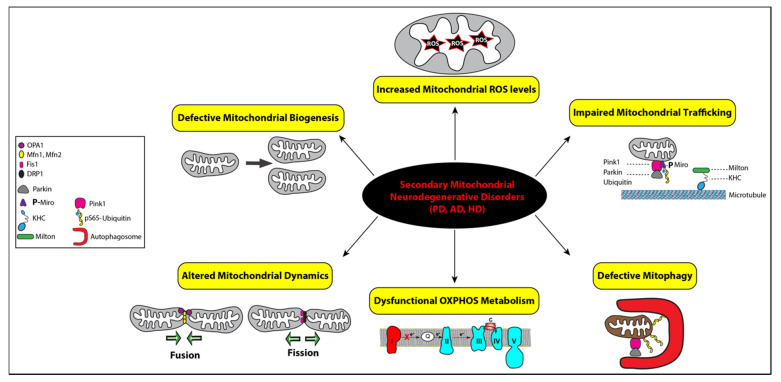
Functional link between mitochondrial dysfunctions and the pathogenesis of secondary mitochondrial neurodegenerative diseases. Mitochondrial homeostasis is regulated by four distinct pathways: biogenesis, dynamics, trafficking, and mitophagy, while mitochondrial bioenergetics is impaired by defective OXPHOS indicated with complex I in red to illustrate its deficiency and increased levels of reactive oxygen species (ROS), represented by a star symbol. The key regulators of mitochondrial fusion (OPA1, Mfn1, and Mfn2) and fission (Fis1 and DRP1) are illustrated with symbols, as detailed in the inset. Impaired mitochondrial trafficking is shown with detached mitochondrion from microtubules via the heavy kinesin chain and the Miro–Milton adaptor complex and ubiquitin. All the corresponding symbols are shown in the inset. Mitophagy is represented with the assistance of autophagosomes (red symbol) engulfing a ubiquitinated mitochondrion that was promoted by Parkin after its recruitment by PINK1 (pink symbol). Mitophagy is impaired in secondary mitochondrial neurodegenerative disorders, Alzheimer’s disease (AD), Parkinson’s disease (PD), and Huntington’s disease (HD).

Secondary mitochondrial diseases can be caused by mutations in genes not encoding OXPHOS subunits, environmental stressors, and/or aging, which result in declined mitochondrial functions [35]. Therefore, impaired mitochondrial dynamics and bioenergetics are major hallmarks of secondary mitochondrial neurodegenerative diseases, such as Parkinson’s disease (PD), Alzheimer’s disease (AD), and Huntington’s disease (HD) (Figure 3) [52].

PD is clinically characterized by tremor, rigidity, bradykinesia due to loss of dopaminergic neurons in the substantia nigra of the brain. It has been well demonstrated that exposure to pesticides and the toxin MPTP (1-methyl-4-phenyl-1,2,3,6-tetrahydropyridine), a prodrug to the neurotoxin MPP^+^, causes parkinsonism by selectively inhibits the activity of complex I in dopaminergic neurons resulting in complex I deficiency (Figure 3) [53]. Although the majority of PD cases are sporadic, about 10–15% of patients affected with early-onset parkinsonism have a familial history. These familial cases are linked to pathogenic variants mapping in several nuclear genes, such as the *SNCA, PINK1, Parkin,* and *LRRK2* genes, all causing complex I deficiency and mitochondrial dysfunction [54]. The missense variant (*A53T*) in the α-synuclein gene (*SNCA*) locus was the first reported genetic cause of familial PD [55]. Other point mutations and multiplication of the SNCA locus have since been linked to PD [56,57,58]. Cytoplasmic inclusions of aggregated α-synuclein protein, namely Lewy bodies, are also the main pathological hallmark of PD [59]. Alpha-synuclein protein was shown to interact with mitochondria and subsequently disrupt mitochondrial morphology, but their precise relationship remains unclear [60,61]. When mitochondria are damaged, PINK1 accumulates on the outer mitochondrial membrane (OMM) and phosphorylates ubiquitin and Parkin at serine 65 (pS65-Ub), triggering the selective autophagic removal of the damaged mitochondria by mitophagy (Figure 3) [62,63]. Loss-of-function mutations in the genes *PINK1* and *Parkin* impair the mitochondrial quality control system, leading to the accumulation of damaged mitochondria and, eventually, PD pathogenesis [64]. Mutations in the *LRRK2* gene alter autophagy, vesicular trafficking, and mitochondrial calcium homeostasis by modulating the autophagy-lysosome pathway [65]. The LRRK2 protein facilitates mitophagy by removing Miro, an OMM protein that anchors mitochondria to microtubules for mitochondrial mobility (Figure 3) [66,67]. PINK1 was also found to phosphorylate Miro to activate the proteasomal degradation of Miro [68]. Studies have also linked mitochondrial dysfunction and chronic inflammation in PD due to damaged mitochondria releasing mtDNA molecules to trigger inflammatory pathways [69]. A recent study from Youle’s group demonstrated the first direct link, where mitochondrial stress in mice lacking PINK1 or Parkin activates cGAS–STING inflammatory pathway and PD-like phenotype [70].

Mitochondrial dysfunction also plays an important role in AD [71]. Pathological hallmarks of AD include deposition of amyloid β plaques and neurofibrillary tangles of phosphorylated tau protein. Amyloid β and phosphorylated tau affect mitochondrial function by interfering with the ETC and inducing mitophagy, which leads to energy defect and loss of mitochondria [72,73,74]. Another major drive of AD progression is an oxidative imbalance, which can be induced by ungoverned leakage of ROS from dysfunctional mitochondria (Figure 3) [75,76,77]. Signs of oxidative damage were detected in autoptic brains from AD patients and AD transgenic mice [24,25]. However, what causes mitochondrial dysfunctions in AD remains elusive. Like PD, most AD cases are sporadic. Nevertheless, the heritability of AD genetic risks have been demonstrated in 56–79% of late-onset AD and over 90% for early-onset AD, which are caused by mutations in the *APP* gene encoding the amyloid precursor protein and the *PSEN1* gene encoding presenilin-1 [78,79,80,81,82]. According to the amyloid cascade hypothesis, neuronal deposition of the amyloid β peptide initiates a cascade of events leading to AD pathogenesis [80]. In AD brain tissues, amyloid β monomers and oligomers were shown to interact with the dynamin-related protein 1 (Drp1), a key mitochondrial fission regulator, thereby altering mitochondrial dynamics via an elusive mechanism (Figure 3) [83]. However, recent failures in amyloid β-based clinical trials have underscored the limitation of the amyloid cascade hypothesis given that mutations in the *APP* and *PSEN1* genes could only explain less than 1% of the AD cases [81,84,85,86]. To identify genetic mutations associated with AD, genome-wide association studies have brought to light over 50 risk-loci, many related to mitochondrial functions, such as the *PPARGC1A* and *NDUFAF7* genes [80,87]. The *PPARGC1A* gene encodes the peroxisome proliferator-activated receptor gamma coactivator 1-alpha (PGC1α), a transcriptional coactivator known to regulate mitochondrial energy metabolism [88]. The *NDUFAF7* gene encodes the NADH dehydrogenase complex I assembly factor 7 protein that assembles and stabilizes the mitochondrial complex I.

The pathogenesis of Huntington’s disease (HD) is characterized by the loss of GABAergic medium spiny neurons in the striatum [89]. HD is mainly caused by an autosomal dominant mutation in the *Interesting Transcript 15* (*IT15*) gene encoding the huntingtin (Htt) protein. The mutated Htt protein can directly interact with the OMM, triggering the loss of mitochondrial membrane potential and permeability [90]. Mutant Htt was also shown to interact with transcription regulators, p53 and PGC-1α, to induce transcriptional dysregulation [91,92]. Increased p53 protein level has been detected in several pathological HD cellular paradigms, murine models, and the brain of HD patients [93]. Upregulation of the p53 protein facilitates the expression of key proapoptotic members of the Bcl2-family, most notably the mitochondrial proteins Bax, PUMA, NOXA, and P53AIP, which results in mitochondrial depolarization and subsequently apoptosis [94,95].

## 3. MS-Based Metabolomics to Study Mitochondrial Disease

### 3.1. Introduction of MS-Based Metabolomics

Metabolomics is the systematic study of small molecule metabolites extracted from a biological sample, such as cells, tissues, and biofluids. Because metabolites represent the downstream result of endogenous genetic/protein regulations and exogenous influences, metabolomics is recognized to be closest to the phenotype compared to genomics, transcriptomics, and proteomics [96]. Due to the diverse classes of metabolites and many unannotated small molecules, identifying all metabolites simultaneously from a given sample is not yet possible. However, mass spectrometry (MS) has become an avant-garde technology for metabolomic studies with its unbeaten high-throughput, sensitivity, specificity, and quantitative accuracy. Thousands of metabolites can be identified and quantified using MS-based analytical platforms coupled with bioinformatics tools to study metabolic perturbations in human diseases.

**Figure 4:**
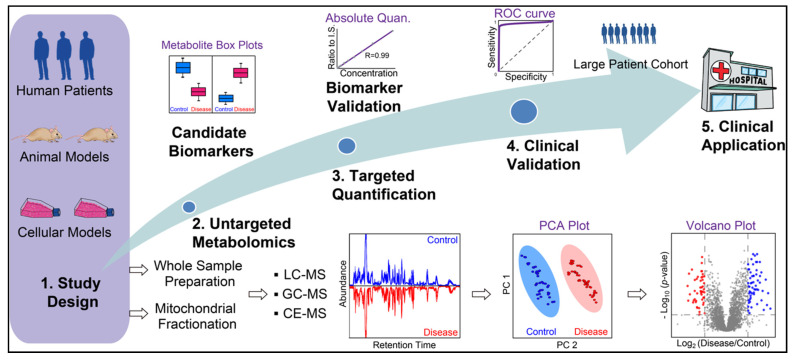
General workflow of MS-based metabolite biomarker discovery for mitochondrial diseases. Whole sample preparation (cell, tissue, and biofluids) and mitochondrial fractionation have been used for metabolomics to study mitochondrial diseases. Untargeted metabolomics is conducted to identify candidate biomarkers, which are then validated by targeted absolute quantification. Next, biomarkers need to be clinically validated with preferably a large patient cohort to examine their sensitivity and specificity prior to their clinical applications.

Metabolomics can be categorized into untargeted and targeted strategies. The untargeted approach aims to obtain the global metabolic profile of a biological system, which has been widely applied to disease biomarker discovery (Figure 4) [97,98,99,100,101,102]. Biofluids from patients, such as blood, urine, and cerebrospinal fluid (CSF), can reflect disease phenotypes and are common sample sources for metabolomics and biomarker discovery. For example, MELAS patients exhibit elevated lactate levels in the blood and/or CSF during lactic acidosis and stroke-like episodes [103,104]. CSF most directly reflects metabolic homeostasis of the central nervous system as it circulates within the ventricular brain system, yet, the collection process through lumbar puncture is invasive and painful [101]. On the other hand, blood or urine is less invasive and easier to obtain, but the metabolic profile can be influenced by the gut microbiome and changes in the whole-body metabolism [105]. Besides patient samples, cellular and animal models are often used to investigate the molecular mechanisms underlying mitochondrial diseases. Primary patient-derived cells and human cell lines are most accessible and quickly available for genome editing to model disease-relevant mutations in immortalized lymphoblastoid cells, cybrid cells, HeLa cells, HEK293 cells, stem cells, and stem cell-derived neurons [106,107,108]. Animal models, such as engineered mice, *Drosophila,* and *Caenorhabditis elegans,* provide in vivo platforms to study the underlying pathogenic mechanisms but fail to recapitulate the clinical symptoms of a specific syndrome [109]. This is a current challenge in the field of primary mitochondrial diseases. Therefore, integrating and validating the molecular changes in multiple disease models and patients is crucial to identify bona fide disease biomarkers and valuable drug targets to develop disease-modifying therapies for human diseases. Bridging bedside to bench and back to bedside is critical for advancing the field of neurotherapeutics for mitochondrial diseases.

For complex biological samples, various separation platforms can be coupled to high-resolution accurate mass (HRAM) MS instruments, such as liquid chromatography (LC), gas chromatography (GC), and capillary electrophoresis (CE) (Figure 4) [110,111,112]. With these hyphenated MS platforms, thousands of metabolites can be identified and quantified with minimum sample materials. Major challenges for untargeted metabolomics include the large-scale and multidimensional dataset as well as an incomplete metabolite library for identification. To facilitate the discovery of metabolite biomarkers, machine learning algorithms have been used in recent years to analyze metabolomics data and assess the sensitivity and selectivity of metabolite biomarkers [113,114]. In contrast to the untargeted approach, targeted metabolomics solely focuses on a handful of metabolites for confident identification and accurate measurement, which often requires standard metabolite compounds for absolute quantification. Stable heavy isotope-labeled metabolite standards can be spiked into samples as internal standards to improve the quantification accuracy and specificity with targeted MS methods but are often expensive and sometimes not commercially available. Targeted metabolomics can be conducted following untargeted metabolomics to verify the changes of key metabolites and candidate biomarkers (Figure 4) [115]. In recent years, MS imaging also gained increased popularity to examine the spatial distribution of metabolites in tissue slices from animal models and patient biopsies [116,117,118,119,120]. MS imaging can also be combined with single-cell analysis with an ultrahigh spatial resolution to identify tissue/region-specific phenotypes that characterize mitochondrial diseases [121,122]. The spatial distribution of neurotransmitters from brain tissues can also be obtained with various MS imaging techniques [123,124,125,126].

### 3.2. Mitochondrial Metabolomics

Mitochondria function as hubs for many metabolic pathways. Therefore, mitochondrial diseases often involve perturbations at the metabolite level, such as amino acids, neurotransmitters, fatty acids, lipids, organic acids, and metabolites in the TCA cycle, glycolysis, fatty acid oxidation, and other key metabolic pathways [97,127,128,129]. Mitochondrial metabolomic studies can be conducted with two distinct strategies: (1) whole-cells, tissues, or biofluid-based metabolomics followed by metabolic pathway/enrichment analysis to tease out mitochondrial metabolites; and (2) isolation of intact mitochondrial fractions followed by metabolomics (Figure 4). Since metabolic pathways are localized in different compartments of eukaryotic cells, post-data metabolic pathway/enrichment analysis can assign identified metabolites into the mitochondrial compartment but also suffers from the high abundant interferences from cytosolic metabolites and metabolite interactions from different organelles [130,131]. Alternatively, intact mitochondria can be isolated from fresh cells or tissues to improve specificity and reduce interferences to analyze mitochondrial metabolites. Differential centrifugation and immunopurification (IP) are the two most common ways to isolate intact mitochondria [132,133]. The differential centrifugation method has been used since 1948 and paved the path for many subsequent mitochondrial studies [134]. However, differential centrifuge often results in impure mitochondrial fraction with interferences from other organelles, such as lysosomes and peroxisomes. It involves time-consuming steps of centrifugations and washes to remove impurities, during which the mitochondrial metabolite profiles can be altered due to the residual activity of enzymes and fast diffusion of small molecules [135,136]. In recent years, rapid and specific IP methods have been developed to isolate intact mitochondria from cells and tissues. The Sabatini group pioneered this approach by tagging OMM protein with the human influenza hemagglutinin (HA) epitope to achieve efficient mitochondrial enrichment with anti-HA antibody-coated beads in both cells and mouse tissues [137,138,139]. However, the IP method requires genetic engineering of cells and animals with epitope-tagged mitochondria, which is not feasible for patient-derived samples.

Stable-isotope metabolic flux analysis (MFA) is another approach to study mitochondrial metabolites while reducing interferences from other cellular compartments. Stable isotopic tracers, such as ^2^H, ^13^C, ^15^ N, ^18^O, can be introduced into the metabolic pathway under physiological conditions to quantify their spatial and temporal dynamics with MS-based platforms. The most common approach involves directly feeding cells with heavy isotope-labeled nutrients, such as glucose and amino acids, followed by MS-based metabolomics [140,141]. MFA can be combined with subcellular fractionation to examine the metabolic fluxes in mitochondria, cytosol and other organelles to provide molecular insights into dynamic mitochondrial metabolism [142].

### 3.3. MS-Based Metabolomics in Primary Mitochondrial Diseases

There are two major aims of MS-based metabolomics studies: to discover disease biomarkers and to unravel their pathogenic and molecular mechanisms underlying the disease. Herein, we discuss key biosignatures and metabolic pathways resulted from MS-based metabolomics studies, as well as their clinical and functional insights for primary and secondary mitochondrial diseases. As the hallmark of primary mitochondrial disease, impaired OXPHOS not only causes ATP shortage but also disturbs several metabolic pathways, such as TCA cycle, glycolysis, fatty acid/phospholipid metabolism, acylcarnitine metabolism, and one-carbon metabolism [109,143,144,145,146]. Abnormalities in glutamate, pyruvate, lactate, acylcarnitines, fatty acids, and amino acids are well recognized, although large cohort validation still needed to be exclusive from other mitochondrial diseases [147,148,149].

Redox imbalance is the most instant and direct consequence of uncoupled ETC besides dropped ATP level. In mitochondria, electrons are diverted in the form of nicotinamide nucleotides and flavin coenzymes. Under basal conditions, functional complex I and II rapidly consume NADH and FADH2 and pass on electrons to reduce oxygen to water in complex IV. In the context of OXPHOS deficit, however, NADH and FADH2 accumulate due to complex I (and/or complex II) defect, causing redox imbalance. Imbalanced redox cofactors regulate over 200 enzymes, such as citrate synthase, isocitrate synthase, α-ketoglutarate dehydrogenase, etc., [150]. In MELAS syndrome, for example, upregulated NADH level inhibits pyruvate dehydrogenase and prevents the conversion of pyruvate to acetyl-CoA, causing pyruvate accumulation. Meanwhile, increased NADH level concurrently activates lactate dehydrogenase, which shunts the accumulated pyruvate to lactate, leading to an elevated lactate level [109]. This rationalizes the pathogenesis of lactic acidosis in MELAS patients.

Interestingly, increasing the pyruvate/lactate ratio by supplying pyruvate has shown potential to treat myopathy in mitochondrial diseases [151]. The metabolic effects of pyruvate treatment were uncovered by MS-based metabolomic study in cybrid cells harboring MELAS variants [152]. Significantly decreased NAD/NADH ratio and increased lactate/pyruvate ratio were detected in MELAS cybrid cells, suggesting a NAD shortage and tendency toward lactic acidosis. In patient-fibroblast-derived induced pluripotent stem cells (iPSCs), pyruvate, lactic acid, malic acid, palmitic acid, stearic acid were identified as candidate biomarkers for m.10191T > C pathogenic variant associated with the MELAS/Leigh overlap syndrome [153]. Decreased NAD/NADH ratio and pyruvate/lactate ratio have been commonly used to indicate redox imbalance and respiratory enzyme deficiency in key energy pathways, such as glycolysis and TCA cycle [109,154,155,156,157,158,159].

TCA cycle is particularly vulnerable to OXPHOS complex deficit because complexes I and II facilitate two of the TCA intermediary steps (i.e., the conversion of α-ketoglutarate to succinyl-CoA, and succinate to fumarate, respectively). The impact of OXPHOS deficit on TCA intermediates was thoroughly reviewed recently [2]. Many pathogenic nuclear and mitochondrial variants associated with a specific primary mitochondrial disease cause complex I or II deficit, such as *Ndufs4*, *MTND3,* and *MTND6* [160,161]. MS-based metabolomics has been instrumental in deciphering the metabolic alterations linked to complex I deficit in various cellular paradigms, *C. elegans* and mouse models, which revealed downregulation of key metabolites in metabolic pathways, including the TCA cycle glycolytic metabolism, pyruvate metabolism, and glutathione metabolism [147,162,163,164,165].

Oxidative stress is another consequence of OXPHOS deficiency that dramatically alters cellular metabolism. Uncoupled electron flow compromises the consumption of oxygen in the mitochondrial matrix, resulting in elevated levels of dissolved oxygen, thereby triggering cellular oxidative stress [166]. It is now recognized that leakage of reactive oxygen species (ROS) is an adaptive metabolic response, also referred to as innate immunity, to infection, diseased state, toxin, or nutritional imbalance [166]. ROS signaling oxidizes thiol groups in metabolites, such as cysteine and glutathione, to form disulfide bonds (e.g., glutathione disulfide (GSSG)) [150]. Iron–sulfur clusters and redox-responsive sites in proteins, such as NADPH oxidases, are also oxidized [167]. Therefore, mitochondrial release of ROS constitutes a signal that has three objectives: (1) to eliminate nutrient waste via production arrest; (2) to stiffen cell membrane for halting the spread of damage; and (3) to ground potential pathogens [166]. Oxidative stress also disrupts fatty acid metabolism, essentially by the fatty acid β-oxidation (FAO) pathway, which provides a powerful energy source in the form of mitochondrial ATP in cells in high-energy demanding organs including the brain, heart, and skeletal muscles (Figure 1). Diseases caused by specific monogenic variants targeting fatty acid transport or FAO are classified as mitochondrial fatty acid β-oxidation disorders (FAODs) [168]. The role of mitochondrial dysfunction and oxidative stress in these diseases was detailed in a recent review [168]. To undergo FAO in the mitochondrial matrix, long-chain fatty acids are actively transported across the outer mitochondrial membrane using the mitochondrial carnitine shuttle and the enzyme carnitine palmitoyltransferase I for converting to long-chain acylcarnitines. In the intermembrane space, acylcarnitines cross the inner mitochondrial membrane via carnitine-acylcarnitine translocase to reach the mitochondrial matrix, where they are converted to acyl-CoAs prior to undergoing FAO. When FAO is defective, the reverse process is activated with acyl-CoAs converted into acylcarnitines and expelled into cytosol. Acylcarnitines have served as candidate biomarkers for inborn error of fatty acids metabolism with neonatal screening of blood, facilitating the diagnosis of FAODs [168,169,170].

### 3.4. MS-Based Metabolomics in Secondary Mitochondrial Disease

Secondary mitochondrial diseases could be more complex than primary mitochondrial diseases. Besides nuclear and mitochondrial genetic determinants, non-genetic factors like age, environmental factors, lifestyle, and nutrition, play an active role in the etiopathogenesis of secondary mitochondrial neurodegenerative diseases. These non-genetic risk factors magnify the challenges of biomarker discovery. In the case of Alzheimer’s disease (AD), aging is the major risk factor with a doubling rate of incidence every five years after the age of 60 [171]. AD and aging also share several common metabolic changes in key metabolic pathways, such as the TCA cycle, the arginine biosynthesis pathway, the proline metabolic pathway and purine metabolism [172].

Traditional biomarker research for secondary mitochondrial diseases mainly focused on measuring individual neurotransmitters, such as dopamine, serotonin, epinephrine, γ-aminobutyric acid (GABA), epinephrine, and norepinephrine. However, recent studies revealed the multifactorial nature of secondary diseases and the urgency to identify reliable biomarkers of secondary mitochondrial diseases for early diagnosis, accurate prognosis, and drug design [35]. While controversial results often arise in metabolomics studies pertaining to secondary mitochondrial diseases, consensus conclusions may be drawn when examining metabolic pathways as a whole instead of individual metabolites. High-throughput MS-based metabolomic studies have allowed mapping thousands of metabolites into key biological processes, including neurotransmission, antioxidation, anti-inflammation, bioenergetics, and to understand how their perturbations are linked to secondary mitochondrial diseases [97,173].

An altered neurotransmission system is another cardinal feature of secondary mitochondrial diseases. The monoaminergic system includes the regulation of dopamine, norepinephrine, serotonin, and other associated metabolites in the central nervous system (CNS), which modulate neurocognition, memory, and neuropsychiatric symptoms [174]. The catecholamines, dopamine and norepinephrine, are the two major neurotransmitters in CNS. The dopaminergic pathway serves as a major target for biomarker discovery of neurodegenerative diseases. Significant depletion of dopaminergic neurotransmission has been reported in PD patients and is closely associated with cognitive decline in AD and Huntington Disease (HD) [175,176,177]. The dopaminergic neuronal loss in the substantia nigra pars compacta (SNpc) of patients with PD involves mitochondria-mediated selective apoptosis [178]. Interestingly, dopaminergic neuronal loss linked to AD exclusively occurs in the ventral tegmental area (VTA) by a mechanism that remains elusive [179]. Precursors of dopamine, including L-tyrosine, L-3,4-dihydroxyphenylalanine (L-DOPA), and 3,4-dihydroxyphenylacetic acid (DOPAC), were also identified as candidate biomarkers in MS-metabolomics for secondary mitochondrial diseases [180,181]. Norepinephrine scavenges oxidative stress by producing antioxidants, such as glutathione. In AD, norepinephrine depletion due to loss of norepinephrinergic neurons in the LC contributes to oxidative damage and mitochondrial membrane permeabilization. However, excess norepinephrine also exhibits neurotoxic effects by elevating cytosolic and mitochondrial oxidative stress through an understudied mechanism [182]. Serotonin (5-hydroxytryptamine, or 5-HT) is a metabolic product of tryptophan. Serotoninergic metabolism is altered in AD due to mitochondrial membrane permeabilization and mitochondria-mediated caspase-dependent apoptosis. This leads to ROS accumulation, further contributing to AD progression [183]. In addition, serotonin is associated with melatonin production, which acts as both an antioxidant and anti-inflammatory factor. In AD models, supplementing melatonin rescued OXPHOS defect [184]. As monoaminergic neurotransmitters are derived from aromatic amino acids, alternations in amino acids metabolisms are commonly reported in secondary mitochondrial diseases. Perturbation in tyrosine and tryptophan metabolism and other neurotransmitter pathways (GABA, glycine, aspartic acid, and glutamic acid) was reported in patients with AD using the LC-MS or CE-MS platform [101,185,186,187,188,189].

Inflammation is a considerable driving force in secondary mitochondrial diseases, with cholinergic (acetylcholine and its metabolites) and purinergic neurotransmitters playing instrumental roles. Acetylcholine is the first discovered neurotransmitter [190] and is linked to various physiological processes, including cognitive function, arousal, blood pressure and anti-inflammatory process [191,192]. Choline, a precursor of acetylcholine as well as glycerophosphocholine and phosphocholine, has been identified as a potential AD biomarker in transgenic mouse models and cerebrospinal fluid (CSF) from patients with AD [193,194,195]. On the other hand, purine metabolism is linked to pro-inflammatory cytokine production as part of the neuroinflammation pathway [196]. Purinergic neurotransmitters include purines, such as adenosine, ATP, and AMP. The discovery of ATP as a neurotransmitter and its role in purinergic signaling in inflammation have been thoroughly reviewed elsewhere [197,198]. Sensitive quantification of AMP, ADP, ATP, and cyclic AMP (cAMP) at nanomolar concentrations was achieved using MS platforms [199]. MS imaging also revealed substantial changes in purine metabolism in an AD transgenic mice model with upregulation of the pro-inflammatory biomarker uric acid and downregulation of the anti-oxidant ascorbic acid [200]. In addition, CSF levels of xanthine, a purine compound, and homovanillinic acid, a dopamine catabolite, as well as their ratio, may serve as potential biomarkers for PD [201].

Signs of oxidative stress are commonly found in secondary mitochondrial diseases due to associated mitochondrial dysfunction. Decreased levels of antioxidants, such as uric acid, ascorbic acid, and glutathione, and increased levels of oxidized glutathione were found in PD and HD as a response to oxidative damage [202,203,204,205,206]. Significantly increased levels of 8-hydroxy-2-deoxyguanosine (8-OHdG) and 8-hydroxyguanosine (8-OHG) were detected in patients with PD, both indicators of DNA damage due to ROS damage [207]. Similarly, a reduced level of glutathione scavenges ROS and reduces Aβ-related oxidative stress in AD [208]. In addition, oxidative stress alters the metabolism of fatty acids and lipids. Recent MS-based untargeted metabolomics revealed altered sphingolipids and glycerophospholipids as antioxidative stress responses in PD [209]. Elevated levels of acylcarnitines found in CSF from patients with PD were associated with lipid biosynthesis perturbated by oxidative stress [209].

Considering that CNS mainly relies on aerobic metabolism for energy supply via the mitochondrial OXPHOS pathway, deficiency in metabolic pathways converging toward OXPHOS plays a crucial role in triggering the early pathogenic stages of neurodegeneration. In secondary mitochondrial diseases, impaired OXPHOS pathways lead to similar metabolic consequences than in primary mitochondrial diseases. Decreased glucose metabolism was reported in the early stage of AD preceding symptoms of cognitive deficits [128,129]. Downregulation of glycolysis and TCA cycle was also observed in PD models via MS-based metabolomics [210,211].

### 3.5. Metabolomics in Mitochondrial Disease-Specific Induced Pluripotent Stem Cells (iPSCs)

To circumvent the inaccessible patient’s affected tissues, such as the CNS and heart, patient-derived iPSCs generated from reprogrammed fibroblasts serve as in vitro models to decipher the metabolic consequences of disease-related pathogenic variants in various patient-specific somatic cells as well as to identify candidate lineage-specific biomarkers. In the context of maternally inherited mitochondrial diseases, generating iPSCs is hampered by the bimodal segregation toward a homoplasmic state of WT or mutated mtDNA during the reprogramming of heteroplasmic patient-derived fibroblasts, which is similar to the mtDNA bottleneck occurring during epiblast specification [212,213]. Moreover, the efficiency of fibroblast reprogramming to iPSCs is substantially decreased by specific mtDNA variants, such as the MELAS variant m.3243A > G [214,215]. Furthermore, high heteroplasmy of m.3243A > G impairs the cellular fate-determination process by inhibiting maturation and survival of iPSC-derived cardiac and neuronal lineages [216]. Metabolomic studies using patient-derived iPSCs with the m.10191T > C pathogenic variant causing the MELAS/Leigh overlap syndrome identified several candidate biomarkers, such as pyruvate, lactic acid, malic acid, palmitic acid, and stearic acid [153]. In contrast, Parkin deficiency does not alter neuronal differentiation of iPSC generated from fibroblasts of patients with Parkinson’s disease caused by *PARK2* mutations [217]. A recent LC-MS metabolomic study on iPSC-derived neurons with parkin deficiency due to *PARK2* mutation reveals increased TCA cycle activity resulting in elevated levels of the TCA cycle intermediates citrate, isocitrate, alpha-ketoglutarate, succinate fumarate, malate, and oxaloacetate [218]. This is accompanied by a dysregulated glucose metabolism characterized by elevated levels of intermediates, such as fructose-1,6-bisphosphate, and decreased levels of pyruvate, resulting in a highly elevated level of lactate. Complementary is the dysregulation of carnitine homeostasis, the key to transport medium and long-chain fatty acids through the outer and inner mitochondrial membranes into the mitochondrial matrix for fatty acid oxidation and energy production [219].

## 4. Conclusions and Future Remarks

In recent years, MS-based metabolomics has become a major player to unravel the molecular mechanisms of primary and secondary mitochondrial diseases. Candidate biomarkers obtained from metabolomic studies need to be validated by targeted quantification and biochemistry assays (Figure 4). However, their clinical validation requires a large patient cohort for future clinical applications (Figure 4). Combining these powerful methods results in improved patient care and ultimately personalized medicine.

Despite progress in technology and our understanding of disease processes, current treatments for most mitochondrial neurological diseases remain palliative in nature and fail to halt their progression. Currently, several agents, such as electron transfer chain function supporter (coenzyme-Q10), energy buffer (creatine), and ROS scavengers (vitamin C, vitamin E, lipoic acid, RP103, and EPI-743) are administered to patients to curtail the ATP deficit due to OXPHOS defects [220]. Idebenone, a coenzyme Q10 analog with antioxidant properties and increased permeability to the blood–brain barrier, has been shown to improve visual acuity in some patients affected with LHON [221].

Current drug designs primarily aim to prevent or eliminate protein aggregation, such as α-synuclein in PD or amyloid β in AD [173,222]. However, to the best of our knowledge, no successful cases have been reported. Alternatively, therapeutic strategies that aim to restore disturbed metabolism directly by designing pathway activators or inhibitors may be more effective [173]. Restoring a disturbed redox imbalance, more specifically NADH levels, is an attractive and promising therapeutic avenue in view of the universal significance of NADH accumulation in the pathogenesis of mitochondrial diseases. While ATP shortage reflects the direct consequences of OXPHOS defect, redox imbalance is now believed to have a more profound impact on cellular metabolism than ATP shortage. In some pathological paradigms, ATP shortage from deficient OXPHOS could be compensated by glycolysis. However, removing accumulated NADH in mitochondrial diseases requires consuming alternative electron acceptors, such as pyruvate that modulates downstream pathways [165]. One promising approach to reduce NADH levels entails genetically engineered expression of an NADH oxidase from *Lactobacillus brevis* (*Lb*NOX) that could improve cellular growth due to impaired OXPHOS [223].

An alternative strategy consists of inhibiting other NADH-producing processes. Methylenetetrahydrofolate dehydrogenase 2 (MTHFD2), a metabolic enzyme that facilitates one-carbon metabolism, was found to be upregulated in mitochondrial diseases [224,225]. (Actually, up-regulation of one-carbon metabolism was often found as an early response to mitochondrial defects [143,226].) MTHFD2 oxidizes 5,10-methylene-tetrahydrofolate (THF) to generate the purine precursor 10-formyl-THF and reducing NAD to NADH. After inhibiting mTORC1, MTHFD2 expression is decreased, and one-carbon metabolism level is restored, which prolongs the survival of mice with engineered complex I deficiency [225,227]. A recent MS-based study from Yang et al. revealed that serine catabolism is the primary source of NADH in cells with impaired OXPHOS, and inhibition of mitochondrial serine catabolism could ameliorate NADH buildup and the disease progression in the Leigh syndrome mice model [165].

Mitochondrial diseases predominantly originate from pathogenic gene mutations and their impact cascading down to all levels of the central dogma of molecular biology. Thus, multi-omics approaches, such as genomics, transcriptomics, proteomics, metabolomics, can be combined to achieve a comprehensive understanding and mutual validation of the molecular processes involved in disease pathogenesis. Meanwhile, the development of MS-based metabolomics requires multidisciplinary efforts to reveal its full potential in neurological diseases. Besides the rapid evolution of mass spectrometry, the development of metabolite databases and computational tools for compound identification and data interpretation are in urgent need. Adding MS-based metabolomics to the multi-omics toolkit will benefit the understanding of the molecular mechanisms, biomarker discovery, and drug design for mitochondrial neurodevelopmental and neurodegenerative diseases.

In conclusion, integrating quantitative analyses from distinct multiparametric metabolic, genetic and clinical investigations that include MS-based metabolomics from patient-derived cells, the patient’s functional mitochondrial energy signature, the patient’s genomic and epigenomic landscape, and the patient’s comprehensive clinical phenotypic manifestations will enable to generate a metabotypic map for each patient. This will allow for the precision prescription of therapies rather than the one-size-fits-all approach developed for the “average patient”. Understanding each patient’s mitochondrial metabotype will result in a more meaningful classification of patients for clinical trials than the current one solely based on a specific pathogenic variant.

## Figures and Tables

**Table 1:** The most frequent primary mitochondrial diseases with maternal or autosomal inheritance.

Name	Pattern of Inheritance	Variants	Onset	Key Clinical Features
KSS	Mt	Deletion of 4977 bp of mtDNA	Late childhood	Progressive ophthalmoplegia, pigmentary retinopathy, cerebellar ataxia, cardiac conduction defects
Leigh	Mt or nuclear (AR)	m.10191T > C (ND3) m.10197G > A (ND3) m.13573G > A (ND5) m.14487T > C (ND6) *SURF1* *COX10* *COX15* *SCO2*	Infancy or early childhood	Ataxia, intellectual retardation, hypotonia, motor delay, cardiomyopathy, brainstem dysfunction, and demyelination
LHON	Mt	m.11778G > A (ND4) m.3460G > A (ND1) m.14484T > C (ND6)	Early adulthood	Optic neuropathy with acute or subacute loss of central vision
MELAS	Mt	m.3243A > G (mt-tRNA^Leu(UUR)^) m.1630A > G (mt-tRNA^Val^) m.3697G > A (ND1) m.13514A > G (ND5) m.14453G > A (ND6)	Childhood or early adulthood	Encephalopathy, lactic acidosis, stroke-like episodes, myopathy, seizures, cognitive deficit, recurrent migraines, gastrointestinal dysmotility
MILS	Mt	m.8993T > G (ATPase6) m.4681C > T (ND2) m.135513G > A (ND5)	Infancy or early childhood	Encephalopathy, developmental delay, hypotonia, lactic acidosis, seizures, ataxia, optic atrophy, dysphagia

Abbreviations: AR: autosomal recessive; KSS: Kearns–Sayre syndrome; LHON: Leber’s hereditary optic neuropathy; MELAS: mitochondrial encephalopathy, lactic acidosis and stroke-like episodes; MILS: maternally inherited Leigh syndrome; Mt: maternal; mtDNA: mitochondrial DNA; mt-tRNA: mitochondrial tRNA.
